# A rapid and easy method for the DNA extraction from *Cryptococcus neoformans*

**DOI:** 10.1186/1480-9222-13-5

**Published:** 2011-07-21

**Authors:** Fatma Mseddi, Mohammed Ali Jarboui, Amira Sellami, Hayet Sellami, Ali Ayadi

**Affiliations:** 1 Laboratoire de Biologie Moléculaire Parasitaire et Fongique, Faculté de Médecine de, 3029, Sfax, Tunisia

## Abstract

DNA isolation from *C. neoformans *is difficult due to a thick and resistant capsule. We have optimized a new and rapid DNA isolation method for *Cryptococcus *using a short urea treatment followed by a rapid method using a chelex resin suspension. This procedure is simpler than previously reported methods.

## Introduction

Nucleic acid detection methods such as PCR have become a common tool for *Cryptococcus neoformans *species complex identification and diagnosis. Although PCR amplification can be performed directly from cultures, prior isolation of DNA is often preferred [1,2].

As the DNA extraction process eliminates many unknown interfering substances in the biological material, it plays an important role in ensuring consistent test results. DNA isolation from *C. neoformans *is difficult due to a thick and resistant capsule that is not readily susceptible to lyses.

Therefore, efficiency in the DNA extraction method using phenol, chloroform and isoamylic alcohol requires time and toxic solution manipulation, due to the organic solvents that may be hazardous to the environment and to the technician, and also several washing and centrifugation steps increasing the risk of sample contamination [3]. Several methods have been proposed as an alternative to the use of phenol and chloroform, such as commercial kits for DNA extraction. The use of kits offers a low risk of manipulation and they are faster than conventional protocols, but the amount of DNA recovered from the commercial kits is highly variable [4]

The objective of the present study was to compare four DNA extraction protocols from culture of collected strains from 2005-2009. This article summarizes the results of a comparison of the techniques in regards to good amplification and purity of obtained DNA.

## Materials and methods

### Strains

A total of 150 Tunisian *Cryptococcus *isolates from clinical and environmental strains and the following standard strains representing each serotype of *C. neoformans *H99 (serotype A), JEC 21 (serotype D), the hybrid IHEM 13877 (serotype AD), *C. gattii *Wm 276 (serotype B) and IHEM 4159 (serotype C) were used.

### DNA extraction

Total genomic DNA was extracted from culture (10^8^cells/ml) of *Cryptococcus *strains by means of the following 4 procedures: Protocol A used extraction with lyticase, phenol-chloroform and isoamylic alcohol; Protocol B used extraction with chelex, Protocol C used extraction using reagent kit (MasterPure yeast DNA purification KIT (Epicentre, Madison, USA)) and the new protocol D using urea chelex.

### Protocol A: DNA extraction using lyticase, phenol-chloroform and isoamylic alcohol

The method for DNA extraction from culture by phenol-chloroform and isoamylic alcohol (Sigma-Aldrich, SP, Brazil) consisted of the suspension of cells recovered by centrifugation from 15 ml of a shaken (150 rpm) 18 h YEPD culture in 2 ml of SE (1.2 M sorbitol, o.1 M EDTA PH = 7.5) and following the method described by Shin-ichi [5]

### Protocol B: chelex DNA extraction

DNA was extracted using a rapid method based on thermal shock and the chelation of components other than nucleic acids by using a resin suspension, as previously described [6]

### Protocol C: Extraction by kit (MasterPure yeast DNA purification KIT (Epicentre, Madison, USA))

The DNA extraction with kit (MasterPure yeast DNA purification KIT (Epicentre, Madison, USA)) was performed according to manufacturer instructions.

### Protocol D: new protocol using urea chelex

Procedure includes the following steps.

(i) Cells recovered by centrifugation from 15 ml of a shaken (150 rpm) 18 h YEPD culture were washed once with cold water, incubated 3 h in 2 ml of urea buffer (urea 8 M, NaCl 0.5 M, Tris 20 mM, EDTA 20 mM, SDS 2%, pH 8)

(ii) The pellet resuspended in 300 μl of distilled water in a microcentrifuge tube. A volume of 100 μl of Chelex solution (10% Chelex-100 [Bio-Rad, Hercules, Calif.] in an aqueous solution of 0.1% SDS, 1% Nonidet P-40, and 1% Tween 80) was added. The tubes were incubated at 95°C for 30 min and then on ice for 5 min. DNA was removed from the supernatant after 5 min of centrifugation (10,000 rpm) and stored at -20°C until used.

### DNA quantification

DNA sample concentrations were determined by spectrophotometry at the wave length of 260 nm for the DNA and 280 nm for proteins, and the purity observed using OD 260/OD 280, in NanoDrop equipment (Thermo Fisher Scientific, Wilmington, DE, USA). Concentration results are given in ng/μL, and the DNA purity results are reported as the OD 260/OD 280.

### DNA quality determination

The DNA quality was accessed by electrophoresis and suitability for downstream application in RAPD analysis (Random Amplified polymorphic DNA). The quality of the DNA yielded by each method was determined by electrophoresing a 5 μl sample in a 0.8% TBE-agarose gel, stained with ethidium bromide. To further demonstrate the quality of the extracted DNA.

The RAPD analysis was carried out using primer six (5'-CCCGTCAGCA-3') in a volume of 50 μl. The following cycle conditions were used: initial denaturation at 95°C for five minutes, followed by 45 cycles of denaturation at 95°C for one minute, annealing at 36°C for one minute and amplification at 72°C for two minutes, and a final extension at 72°C for 10 minutes. Amplification products were separated by electrophoresis, on 2% agarose gels in 1 × TBE buffer at 150 V for 2.5 hours and stained with ethidium bromide and then visualized under UV light.

## Results

### Comparative analysis of DNA sample quantification

Comparing DNA sample quantification from culture, we observed that the extraction with Protocol D gave the highest DNA concentration (16, 03 μg/ μl), as compared with Protocols A, B and C (Table 1). Therefore, Protocol A presented a lower yield of DNA (4. 29 μg/ μl) than Protocols B and C (Table 1).

**Table 1:** DNA concentration and purity (OD 260/OD 280) obtained by the four DNA extraction techniques

Protocols	Average DNA concentration DNA (μg/ μl)	OD 260/OD 280
**A**	4.29	1.715
**B**	8.36	1.78
**C**	7.54	1.685
**D**	16.03	1.75

### Comparative analysis of DNA quality on agarose gel

**Figure 1:**
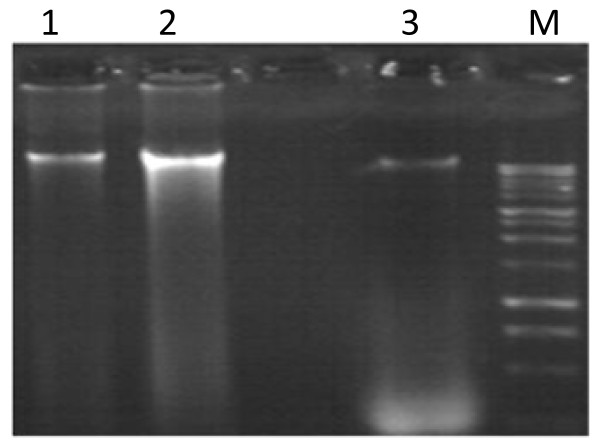
**Electrophoresis of total cellular DNA from an isolate of *Cryptococcus *on 0.8% agarose**. Lane 1: genomic DNA with kit (Master pure), Lane 2: genomic DNA with urea chelex, lane 3 genomic DNA with chelex, M: 1 kilobase DNA leader.

Our results showed a variable yields and quality in DNA across the different extraction methods. Only the new method produced high yields that were of good quality DNA and we have obtained a clear DNA band when 10 μl of the 400 μl DNA preparation was run in an agarose gel (0.8%) and stained with ethidium bromide (Figure 1).

### Comparative Analysis of RAPD Profiles

**Figure 2:**
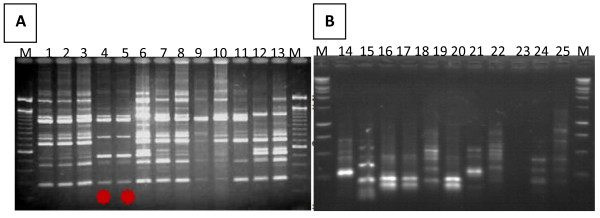
**A comparison of RAPD profiles of *C.neoformans***. Example of environmental and clinical strains of *C. neoformans *amplified by PCR RAPD (lane 1-13 and lane 14-25) **A**- DNA was extracted using urea chelex **B**- DNA was extracted using chelex M: 100 pb DNA ladder.

The results from DNA extractions compared by RAPD PCR generate a profile comprising many bands (Figure 2). The broad range of bands comprising community profiles was visible and intra- and inter-subject variations were readily observed with the new extraction Protocol D. Using the three other protocols (A, B and C), we demonstrated that there were many bands in the upper part of the RAPD gel that were not sufficiently resolved to describe differences (Figure 2).

## Discussion

After experimenting with several DNA purification regimens, we have optimized a new and rapid DNA isolation method for *Cryptococcus *using a short urea treatment, described by Bolano and al. [7] with slide modifications, followed by a rapid method based on thermal shock and the chelation of components, other than nucleic acids, using a resin suspension as previously described [6].

This new method is easier than described by Bolano et al. that incorporate a treatment with urea and bead beating [7]. The whole procedure can be completed within 4 hours. Up to now, we have used the technique to isolate DNA from 150 Tunisian *Cryptococcus *isolates from clinical and environmental strains. In addition, we also have succeeded in generating sufficient DNA from *Cryptococcus *isolates using this rapid method. We have obtained a clear DNA band when 10 μl of the 400 μl DNA preparation was run in an agarose gel (0.8%) and stained with ethidium bromide.

Further, quantity and quality of DNA extract was influenced by the extraction method.

Data from this study indicate that the new DNA extraction method using urea chelex produced high quality DNA that can be amplified using PCR RAPD for comparisons of *C. neoformans *fingerprint profiles. These observations demonstrate that optimal DNA yield from culture is obtained by this method. Higher extraction efficiency allows for better recovery of DNA from an environmental sample resulting in a more comprehensive profile of *C. neoformans*. On the other hand, poor DNA extraction may lead to a poor PCR RAPD profiles. Higher DNA yield also increases recovery of DNA from *C. neoformans *isolate and chances of detecting polymorphism.

This procedure modifies and considerably simplifies previously reported methods for extraction of DNA.

It is likely that this procedure could be applied to the extraction of many other fungal cultures and, possibly, clinical specimens. It provides a rapid, reliable, and low-cost alternative to the existing DNA purification protocols used in research and clinical laboratories. The availability of this DNA extraction procedure for *Cryptococcus *not only would reduce the workload considerably but also would decrease the test turnaround time.

## Competing interests

The authors declare that they have no competing interests.

## Authors' contributions

MF conceived the method, designed and carried out the validation of the study and writes the manuscript. MAJ participated in execution of the study. SA and AA designed the study and contributed to writing the manuscript. All authors contributed to writing the manuscript. All authors read and approved the final manuscript.
